# Supplementary material for: Application of Pinhole Plasma Jet Activated Water against Escherichia coli, Colletotrichum gloeosporioides, and Decontamination of Pesticide Residues on Chili (Capsicum annuum L.)
Source: Foods. 2022 Sep 15;11(18):2859. doi: 10.3390/foods11182859 (PMC9498241; doi:10.3390/foods11182859)
Supplement: Supplementary file 1 [file foods-11-02859-s001.zip › foods-1890625-supplementary.pdf]

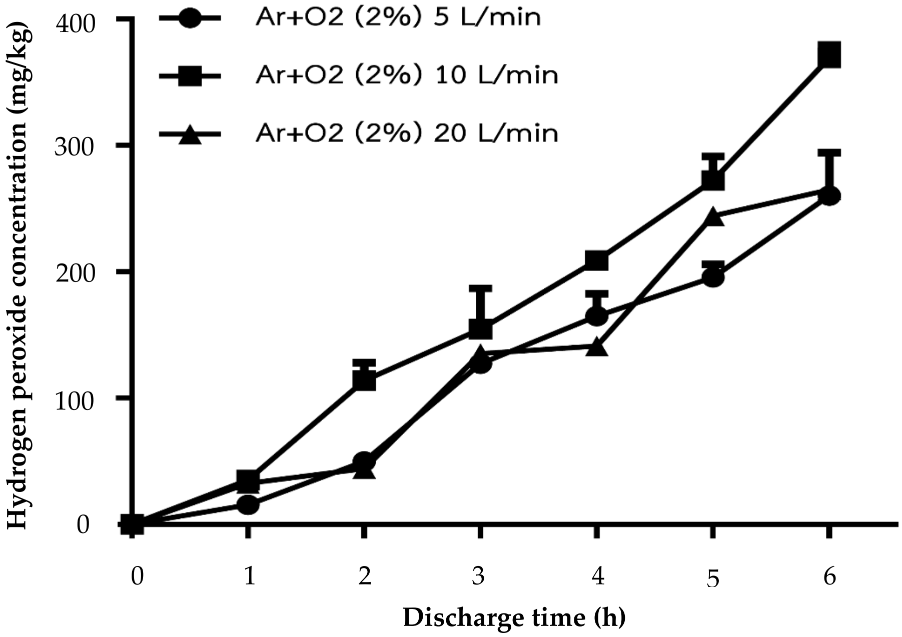

Figure S1. H_2O_2 concentration of PAW produced by different mixture rates of $Ar+O_2$.

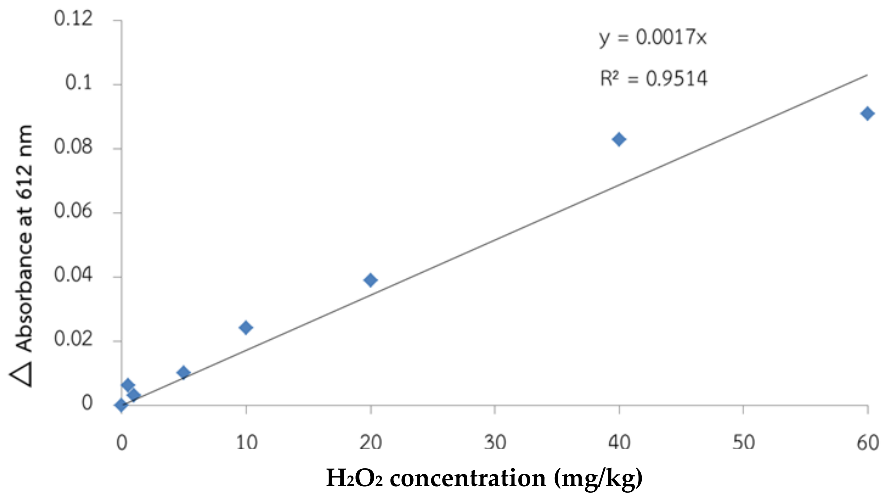

Figure S2. Standard curve of hydrogen peroxide measurement.

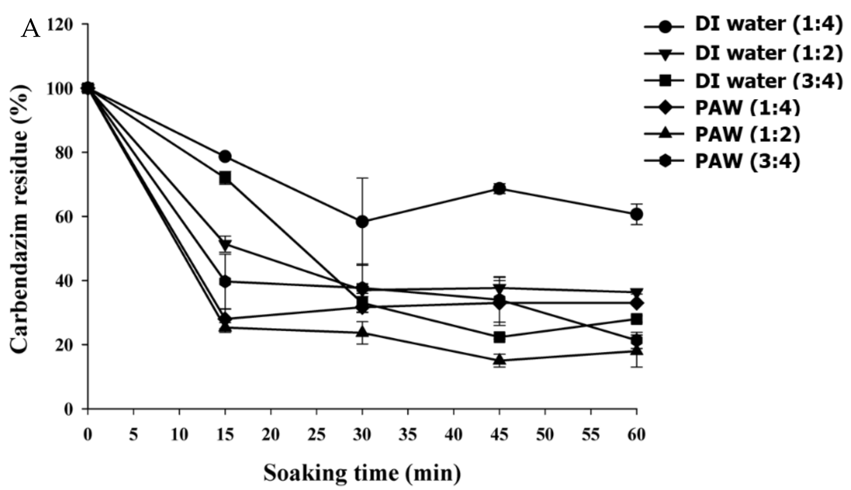

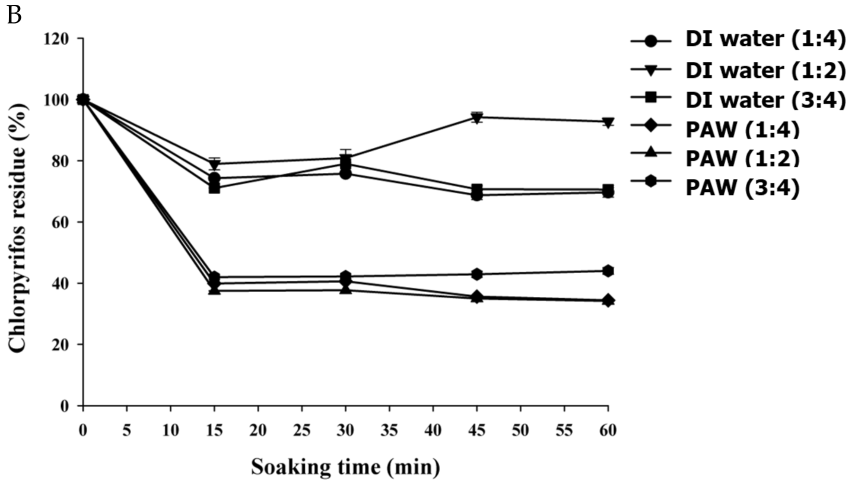

Figure S3. Degradation of (A) carbendazim, and (B) chlorpyrifos residues in chili with different ratios of PAW and DI water as a function of treatment time.
